# Gametocyte clearance in children, from western Kenya, with uncomplicated *Plasmodium falciparum* malaria after artemether–lumefantrine or dihydroartemisinin–piperaquine treatment

**DOI:** 10.1186/s12936-019-3032-3

**Published:** 2019-12-04

**Authors:** Protus Omondi, Marion Burugu, Damaris Matoke-Muhia, Edwin Too, Eva A. Nambati, William Chege, Kelvin B. Musyoka, Kelvin Thiongo, Maureen Otinga, Francis Muregi, Francis Kimani

**Affiliations:** 1 Department of Biochemistry, Microbiology, and Biotechnology, Kenyatta University, P.O Box 43884-00100, Nairobi, Kenya; 2 Centre for Biotechnology Research and Development, Kenya Medical Research Institute, P.O Box 54840-00200, Nairobi, Kenya; 3 Department of Biological Sciences, Mount Kenya University, P.O Box 342-00100, Nairobi, Kenya; 4 Department of Biochemistry, Jomo Kenyatta University of Agriculture and Technology, P.O Box 62000-00200, Nairobi, Kenya

**Keywords:** Artemether–lumefantrine, Dihydroartemisinin/piperaquine, *Plasmodium falciparum* gametocyte

## Abstract

**Background:**

The efficacy and safety of artemether–lumefantrine (AL) and dihydroartemisinin–piperaquine (DP) against asexual parasites population has been documented. However, the effect of these anti-malarials on sexual parasites is still less clear. Gametocyte clearance following treatment is essential for malaria control and elimination efforts; therefore, the study sought to determine trends in gametocyte clearance after AL or DP treatment in children from a malaria-endemic site in Kenya.

**Methods:**

Children aged between 0.5 and 12 years from Busia, western Kenya with uncomplicated *Plasmodium falciparum* malaria were assigned randomly to AL or DP treatment. A total of 334 children were enrolled, and dried blood spot samples were collected for up to 6 weeks after treatment during the peak malaria transmission season in 2016 and preserved. *Plasmodium falciparum* gametocytes were detected by qRT-PCR and gametocyte prevalence, density and mean duration of gametocyte carriage were determined.

**Results:**

At baseline, all the 334 children had positive asexual parasites by microscopy, 12% (40/334) had detectable gametocyte by microscopy, and 83.7% (253/302) children had gametocytes by RT-qPCR. Gametocyte prevalence by RT-qPCR decreased from 85.1% (126/148) at day 0 to 7.04% (5/71) at day 42 in AL group and from 82.4% (127/154) at day 0 to 14.5% (11/74) at day 42 in DP group. The average duration of gametocyte carriage as estimated by qRT-PCR was slightly shorter in the AL group (4.5 days) than in the DP group (5.1 days) but not significantly different (p = 0.301).

**Conclusion:**

The study identifies no significant difference between AL and DP in gametocyte clearance. Gametocytes persisted up to 42 days post treatment in minority of individuals in both treatment arms. A gametocytocidal drug, in combination with artemisinin-based combination therapy, will be useful in blocking malaria transmission more efficiently.

## Background

Artemisinin-based combination therapy (ACT) has been associated with sharp reductions in malaria incidence and malaria transmission intensity in Africa [1]. Despite the rising cases of resistance against commonly used artemisinin-based combinations in the Thai-Cambodia border, South-East Asia [2], ACT still contributes significantly to the decline of malaria disease burden [3, 4]. In Kenya, artemether–lumefantrine (AL) and dihydroartemisinin–piperaquine phosphate (DP) were officially implemented in 2006 as the first-line and second-line drug, respectively, for management of uncomplicated *Plasmodium falciparum* malaria [5]. Adoption of this regimen for the management of uncomplicated malaria has been beneficial because of their transmission-reducing effect [6, 7]. ACT has been associated with a decline in transmission, which is due to the rapid elimination of asexual parasites, and partly due to their effect on gametocytes. Gametocyte clearance after treatment reduces malaria transmission and more importantly prevent the selection and spread of resistant malaria parasites [8]. Generally, ACT is highly effective against asexual stages and immature gametocytes. However, the activity of these anti-malarials against mature gametocytes is limited [9]. Recently, studies have shown that the transmission-reducing effect differs among artemisinin-based combinations [7–10]. However, it is still unclear whether AL and DP are the most appropriate choice for reducing community-wide transmission of *P. falciparum* in a high malaria-endemic setting [9, 11].

Comparative studies on the risk of residual submicroscopic gametocytes after treatment with AL versus DP have reported conflicting results [12, 13]. Some studies indicate an increased risk of gametocyte carriage after AL treatment [1, 12], whereas others suggest that AL has a more pronounced effect on gametocyte carriage compared to DP [7, 9]. Sawa et al. reported a significant impact of AL and DP on post-treatment gametocyte carriage in a different site of western Kenya [7]. With increasing efforts to reduce malaria transmission, it becomes more significant to evaluate the gametocytocidal activity of ACT in various settings to establish the gametocyte clearance trends [14]. Gametocyte dynamics may differ between areas and findings indicate that this may influence the impact of treatment on gametocyte carriage. In light of the observations of declining ACT efficacy in East Africa, re-examination of ACT gametocytocidal efficacy is valuable [15].

Approximately 80% of post-treatment gametocyte densities are often below the detection limit of microscopy [16]. Reverse transcriptase real-time quantitative PCR (qRT-PCR) is a highly sensitive molecular assay for the detection of submicroscopic gametocytes [16]. The qRT-PCR assay was used to detect the female-specific *pfs25* transcript, which is highly expressed in mature female gametocytes (stage V). Female gametocytes *pfs25*-markers are essential because their expression is highly specific to the sexual stages, and their density broadly reflects the overall gametocyte density [14, 17]. Thus, the study sought to determine trends in gametocyte clearance after AL or DP treatment in children from a malaria-endemic setting in Kenya.

## Methods

### Study site

The study covers Matayos sub-county in Busia County, Western Kenya. Busia is an endemic malaria zone in Kenya. In 2017 the disease was reported to be the leading cause of mortality in the county [15]. The area borders Lake Victoria and is located approximately 268 miles (431 km) by road, West of Nairobi, the capital city of Kenya, between latitudes 00° 01′ and 00° 47′ north of the equator. Transmission of malaria increases during long rains that favour breeding of malaria vectors (*Anopheles gambiae* and *Anopheles arabiensis*) resulting in increased intensity of *P. falciparum*.

### Sample collection

The study was part of a larger study on the efficacy of ACT in Kenya that aimed at evaluating the current therapeutic efficacy of artemisinin-based combinations. Samples used in the study were collected from 334 children with uncomplicated *P. falciparum* malaria, aged 6 months to 12 years in Busia, western Kenya. The samples were collected during the peak malaria transmission season from August to November 2016. The study population and treatment procedures were conducted as described elsewhere [17, 18].

Briefly, dried blood samples obtained from eligible children with uncomplicated *P. falciparum* malaria in Matayos Hospital, Busia county, after a random treatment with either AL or DP were used. At initial presentation, the thick smear was prepared from for all children with fever or history of fever within 24 h. Treatment was given under observation, and an additional blood sample was spotted on filter paper (Whatman 903 Protein Saver Card) for those who were included. Levels of haemoglobin were assessed using the Hemocue Hb201 system.

Patients were followed-up on day 1, 2, 3, 7, 14, 21, 28, 42, and any other day before day 42 if any symptoms recurred. On every visit, Giemsa-stained thin and thick blood smear was prepared by laboratory technicians, and both asexual and gametocyte densities were assessed simultaneously by counting against 200 leukocytes and 500 respectively. Samples were considered negative if no parasites were detected in 100 fields (10 × 100 magnification). Dried blood spots were collected on every visit for further molecular investigation.

The dried blood spots were sealed in zip locks bags containing silica gel desiccant and stored in − 20 °C freezer in the Kenya Medical Research Institute (KEMRI) at the Centre for Biotechnology and Research Development (CBRD). For the standards, in vitro culture of stage V gametocytes densities were counted by two independent microscopists and diluted to densities of 10^1^, 10^−1^ and 10^−2^ gametocytes/µl in whole blood. 50 µl of the control samples were stored at 80˚c in RNA stabilizing guanidine isothiocyanate buffer [19]. Large blood spots of the control solutions were aliquoted in 3 to 5 replicates per filter paper, dried overnight and sealed in zip locks bags containing silica gel desiccant.

### *Pfs25* qRT-PCR

Guanidine based extraction method was used to extract RNA from DBS for day 0 and follow-up days as described by Jones et al. [20] with a slight modification. The concentration and purity of RNA was confirmed using nanodrop spectrophotometry and normalized through dilution before subjected to qRT-PCR step. On qRT-PCR step, DNase treatment followed by RT step was done using QuantiTect^®^ Reverse Transcription kit (QIAGEN) according to the manufacture instructions. The reverse-transcription products were then stored on ice before proceeding to quantitative real-time PCR.

For the detection of *P. falciparum* gametocytes present in the cDNA sample, qPCR assay targeting *pfs25* gene was conducted in a 96-well plate on an Exicycler real-time PCR machine (Bioneer) according to the manufacture instructions. *Pfs25* mRNA is highly expressed in mature female gametocytes amplified using *pfs25* primers as described elsewhere [10] using Accupower^®^ 2× GreenStar™ qPCR Master mix (Bioneer). Melting curves were run after every run to evaluate the efficiency of the qRT-PCR. Serial dilution for all the test samples was done and all the samples were assayed in duplicates alongside the standards.

Gametocyte densities were calculated by converting the threshold cycle (Ct) values of test samples to gametocyte density using plate-specific standard curves. The standard curves were made from an in vitro culture of stage V gametocytes [19] (from 2.5 × 10− 2.5 × 10^−3^ gametocytes/µl) as described by Tadesse et al. [21]. The standard curves with a line of best fit was plotted. Samples with estimated densities below 0.02 gametocyte per µl or 2 gametocytes per ml of samples were considered negative [19]. Gametocyte prevalence and density were determined using *pfs25*qRT-PCR on days 0, 7, 14, 21, 28 and 42.

### Statistical analysis

The statistical data analysis was performed using R statistics version 3.5.1 and STATA software version 16 (Stata Corporation, Texas, USA). The data included the parasitological efficacy of AL and DP and submicroscopic gametocyte carriage as the primary and secondary outcomes. The number of individuals included in the qRT-PCR analysis during follow-up was selected randomly. As previously described [7], 50 individuals per treatment arm is enough to detect the difference between arms with a 2-sided type 1 error of 0.05 and a power 95%. Based on the previously estimated average duration of gametocyte carriage of 13.4 ± 7.5 days [21] following AL treatment, and 65% longer duration after DP treatment, a minimum of 70 children per treatment arms were included during follow-up.

Time to treatment failure was defined as described in a previously published efficacy trial [21] and findings between arms compared using logistic regression. Gametocyte densities were calculated on a log scale. A simple deterministic compartmental model as previously published was fitted to data on gametocyte density and prevalence to estimate average circulation time per gametocyte and mean overall duration of gametocyte carriage after treatment for individuals with an adequate clinical response [21]. To quantify the effect of AL and DP treatment on gametocyte carriage, the area under the curve (AUC) for gametocyte clearance by *pfs*25 qPCR was determined as previously described [22] with slight modification and scaled by 42 to represents the AUC per day. Linear regression was used to compare differences in AUC between the two groups. Time to negativity (disappearance of gametocytes) was determined using a Kaplan–Meier estimator and log-rank to test for equality between the functions for AL and DP. Comparisons in between treatment arms in gametocyte prevalence and density were determined using Wilcoxon rank-sum test and Chi square or Fisher’s exact tests.

## Results

**Fig. 1:**
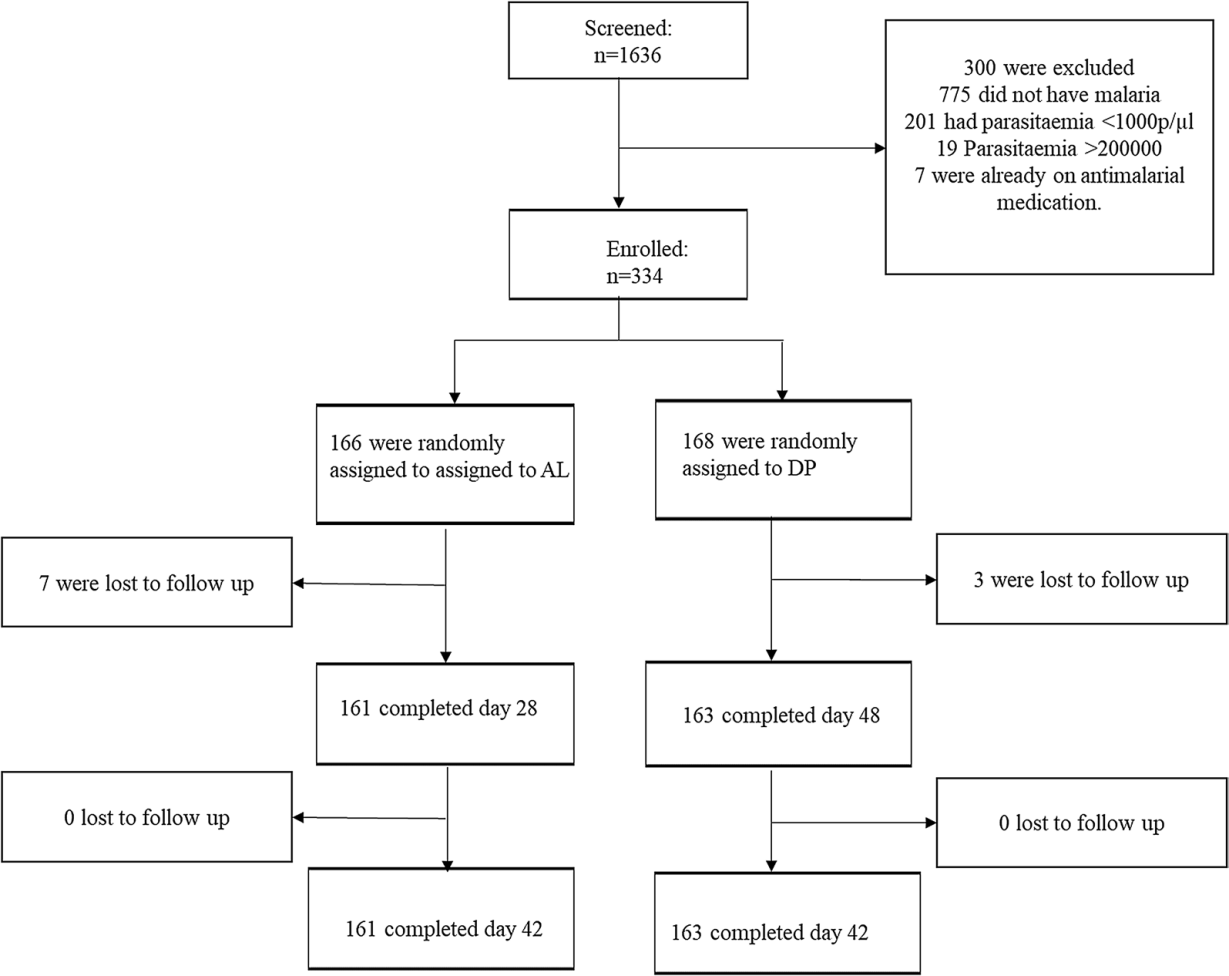
Enrolment flow chart. Representation of patients screening, inclusion and follow-up of the study. **a** Dihydroartemisinin–piperaquine. **b** Artemether–lumefantrine

The 334 children who met the inclusion criteria were enrolled in the study. The participants were aged 0.5 to 12 years where 168 (51.3%) and 166 (49.7%) received treatment with DP and AL, respectively, with no significant difference between the arms (Fig. 1). At enrolment, the geometric mean asexual density was 15,832 parasites/µl (95% confidence interval [CI] 14028–17870) and did not differ between the treatment arms (Table 1). On day two after AL and DP were administered, 6.0% (10/168) in AL arm and 2.4% (4/166) in DP arm had detectable asexual parasites by microscopy. On day three, none of the participants on the AL arm had asexual parasites and only one child in DP arm had asexual parasitaemia of 200 parasites/µl, down from an initial asexual parasite density of 22,000 parasites/µl. By day seven all the children were negative for asexual parasites by microscopy.

**Table 1:** Baseline characteristics of the participants by study arm

Arm	DP	AL	*p* value
N	168	166	0.501
Sex = male (%)^a^	93 (55.4)	90 (54.2)	0.921
Age-months (95% CI)^b^	56.85 (51.34–62.356)	59.93 (54.32–65.54)	0.439
Haemoglobin levels (95% CI)^c^	10.23 (9.95–10.51)	10.45 (10.17–10.73)	0.272
Asexual parasite density, geometric mean at enrolment (95% CI) by Microscopy^b^	12,828.74 (12,828.74–18,135.92)	16,441.55 (13,859.57–19,504.54)	0.564
*Pfs25* qRT-PCT gametocyte prevalence at baseline, % (n/N)^a^	82.4 (127/154)^*^	85.1 (126/148)^*^	0.723
*Pfs25* qRT-PCT gametocyte density at baseline, geometric mean (95% CI)^c^	0.94 (0.62–1.42)	0.85 (0.61–1.19)	0.324

*CI* confidence interval, *IQR* interquartile range, *DP* dihydroartemisinin–piperaquine, *AL* artemether–lumefantrine

*p-value represents the difference between groups. The difference in gametocyte prevalence was tested using Fisher’s exact test

Data included; ^a^prevalence (n/N), ^b^mean (95% CI) or ^c^median (IQR)

*Samples for qRT-PCR analysis at enrolment (DP, n = 154 AL, n = 148 p = 0723.) were selected based on the integrity and availability of samples (slides and DBS) for the different time points of follow-up

On day 42, following treatment 14.1% (23/160) of children in the AL arm were parasite positive by microscopy compared with 21.12% (34/161) in DP arm. This treatment outcome was not statistically associated with treatment arm (p = 0.112), age (p = 0.351) and parasite density (p = 0.677) at enrolment.

### Gametocyte carriage after AL and DP treatment

**Fig. 2:**
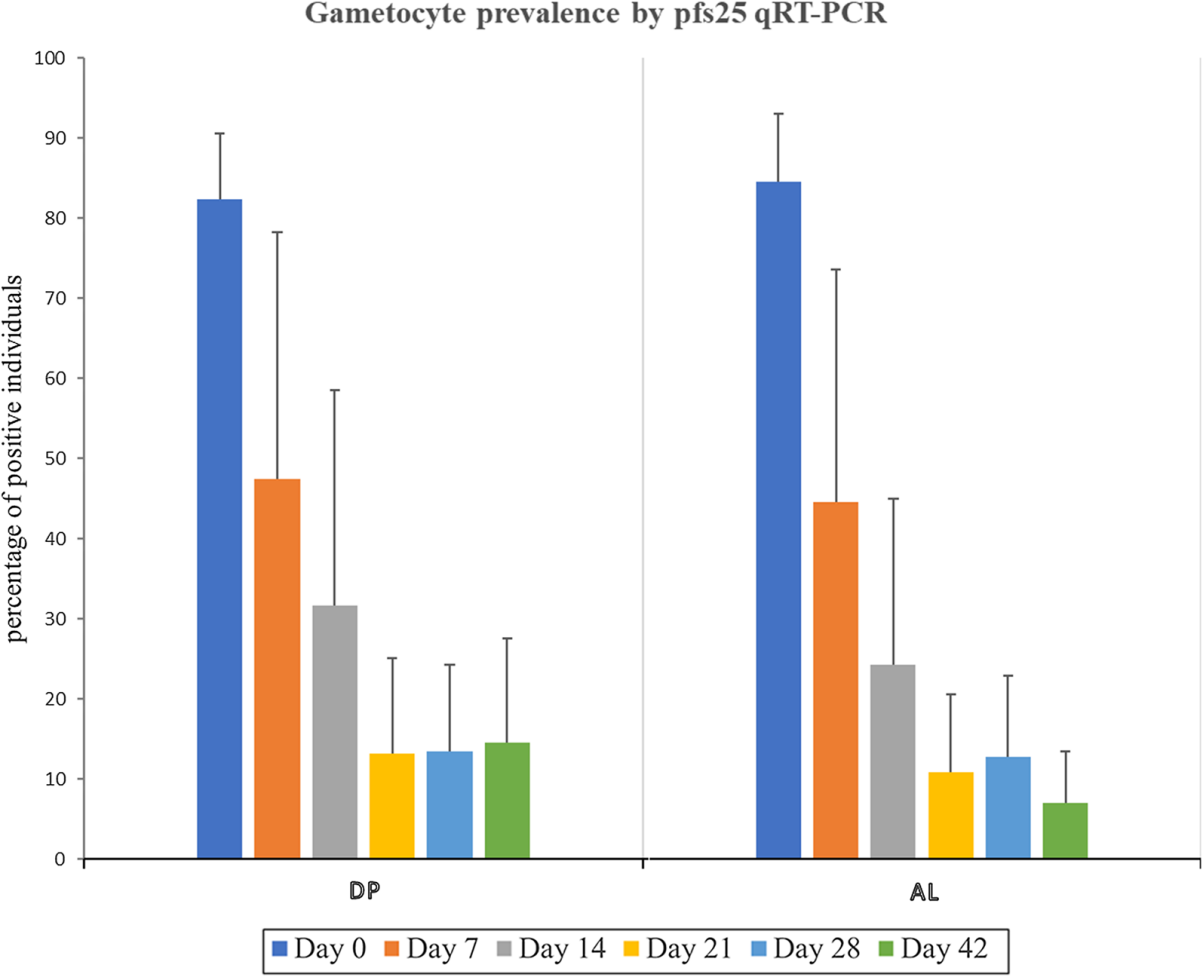
Gametocyte prevalence after AL or DP treatment by Pfs25 qRT-PCR. The error bars indicate the upper limit of the 95% confidence interval for prevalence. *DP* dihydroartemisinin–piperaquine, *AL* artemether–lumefantrine

As expected *Pfs25*qRT-PCR detected more gametocyte positive individuals than microscopy at all time points. Out of the 302 samples, 253 had detectable gametocytes giving a prevalence of 83.7% at enrolment. Overall, *Pfs25*qRT-PCR identified significantly more cases of patients with gametocytes on day 0 (83.7% *versus* 12%; p = 0.02), day 7 (46.0% *vs.* 6.6%; p < 0.001), day 14 (28.6% *vs.* 2%; p < 0.001), day 21 (11.6% *vs*. 1.2%; p < 0.001), 28 (11.4% *vs*. 1%; p = 0.006) and on day 42 (9% *vs.* 5%; p < 0.001) (Fig. 2). Gametocyte prevalence increased on days 28 and 42 of follow-up and was strongly associated with the recurrent parasitaemia on these days.

**Fig. 3:**
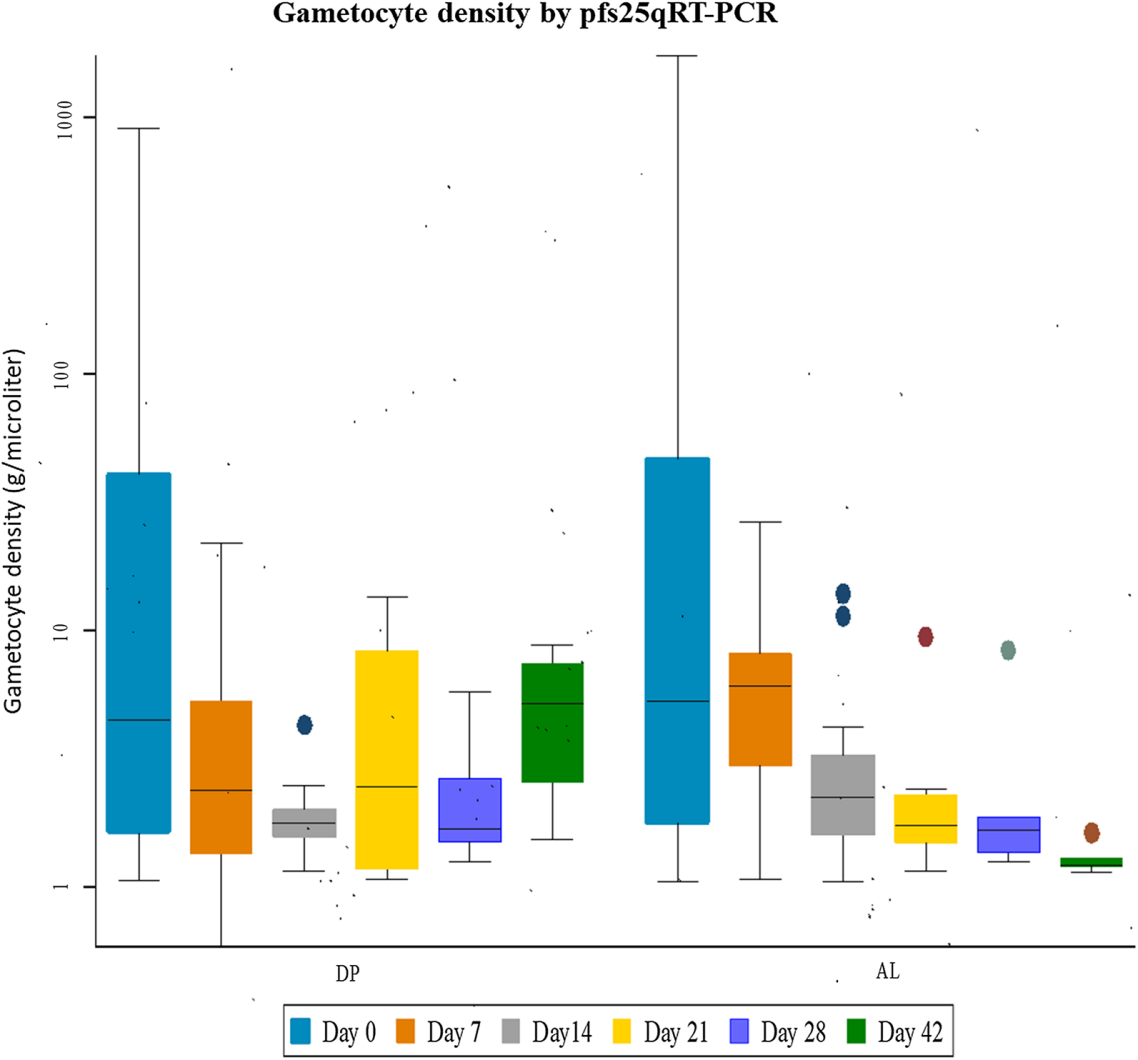
Gametocyte density by Pfs25 qRT-PCR at enrolment and on days 7, 14, 21, 28, 42 after AL or DP treatment. Samples were considered negative if gametocyte levels were < 0.02/µl. Density is presented as median (IQR) for positive individuals only. *DP* dihydroartemisinin–piperaquine, *AL* artemether–lumefantrine

Median gametocyte density among gametocyte positive individuals decreased over time. At baseline gametocyte median density decreased from 1.98 (IQR 0.87–5.30) to 0.42 (IQR 0.22–1.25) on day 42 in AL group and from 2.66 (IQR 0.82–4.15) to 0.37 (IQR 0.16–0.42) in DP group (Fig. 3, Table 2). The mean duration of gametocyte carriage as estimated by qRT-PCR was slightly shorter in AL group (4.5 days, 95% CI 3.70–5.2) than in DP group (5.1 days, 95% CI 4.12–6.10) (p = 0.77) but this difference was not significant. Interestingly, there was a significant difference between the mean circulation time for the AL group (2.21 days, 95% CI 1.71–2.86) compared with DP group (3.14 days, 95% CI 1.97–3.72) (p = 0.007). After adjusting FOR baseline gametocyte density, the AUC showed no significant difference (p = 0.524) between the two groups (Table 3).

**Table 2:** *Plasmodium falciparum* gametocyte density during follow up

Days	Artemether–lumefantrine	Dihydroartemisinin–piperaquine	p value
Day 0	1.98 (0.87–5.30)	2.66 (0.82–4.15)	0.388
Day 7	1.23 (0.15–2.45)	0.92 (0.21–2.85)	0.439
Day 14	0.50 (0.12–1.26)	0.65 (0.34–2.28)	0.457
Day 21	0.34 (0.12–0.76)	0.72 (0.20–1.94)	0.529
Day 28	0.43 (0.32–1.18)	0.53 (0.38–1.84)	0.621
Day 42	0.42 (0.22–1.25)	0.37 (0.16–0.42)	0.355

Data included: gametocyte positive individuals. Median density on day 7, 14, 28 and 42 is not necessarily assessed over the same individuals used to determine gametocyte density at enrolment

*p-value represents difference between groups. Difference in gametocyte density was tested using the Wilcoxon rank-sum test. Median and IQR

**Table 3:** Area under a curve (AUC) and persistence of gametocytaemia after AL and DP treatment

Treatment arm	AUC (g/µl day), median (IQR)	% gametocyte prevalence (n/N)		
		Day 21	Day 28	Day 42
AL	0.56 (0.12–1.26)	10.8 (8/74)	12.7 (9/71)	7.04 (5/71)
DP	0.37 (0.16–0.42)	13.2 (10/76)	13.5 (10/74)	14.5 (11/74)
p-value	0.524	0.77	0.521	0.342

Adjusted for baseline gametocyte density

*IQR* interquartile range, *DP* dihydroartemisinin–piperaquine, *AL* artemether–lumefantrine

**Fig. 4:**
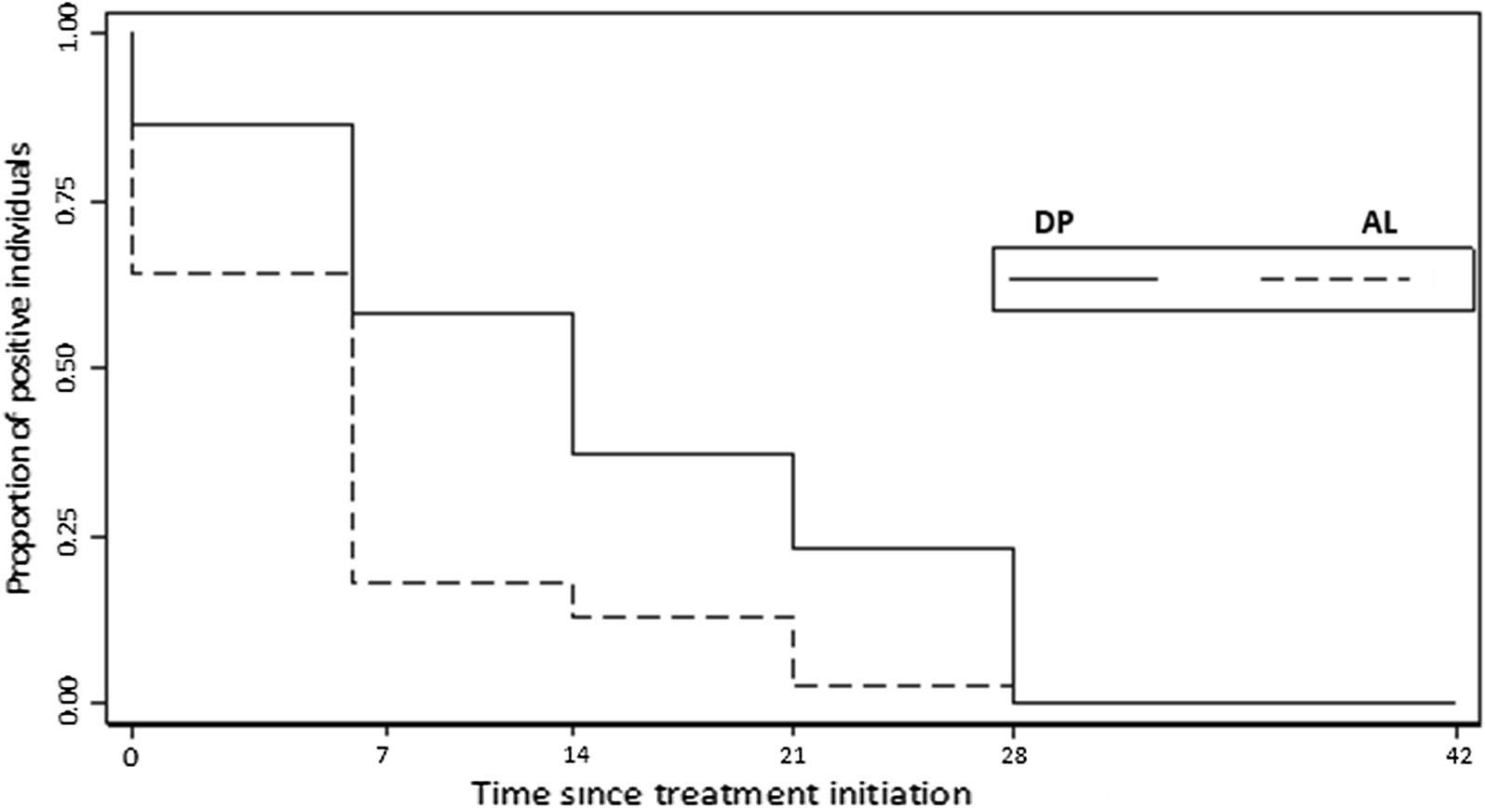
Kaplan-Meier plot of time to the negativity of individuals who were gametocyte positive before either AL or DP treatment. The proportion of gametocyte positive children are shown on the Y-axis and time to complete gametocyte clearance in individuals who were gametocyte positive at enrolment on the X-axis. Time to disappearance was estimated for patients who were positive for gametocyte before treatment only. Artemether–lumefantrine (dashed line n = 43) or dihydroartemisinin–piperaquine (solid line n = 39) in weeks on the X-axis. p = 0.5731. Difference between arms was tested using the log-rank test

On the other hand, survival analysis of data restricted to gametocyte positive individuals by qRT-PCR at enrolment indicated that time to gametocyte clearance was shorter for AL group compared with DP group (p = 0.5731), but this difference was also not significant (Fig. 4).

## Discussion

This study highlights the dynamics of *P. falciparum* gametocytes using a molecular-based method after AL or DP treatment. The study detects no significant difference between AL and DP in gametocyte clearance rate. Gametocyte prevalence in the present study site at enrolment was 83.7% by RT-qPCR compared to 12% by microscopy. This difference is consistent with the previous findings in both symptomatic and asymptomatic cases [10, 22], suggesting the underestimation of gametocyte prevalence by microscopy. During the 5 weeks of follow-up, AL appears to clear *P. falciparum* gametocytes slightly faster than DP but the difference was not significant. The findings on gametocyte circulation time confirm the previous estimates that used sensitive molecular assays [21]. The average duration of gametocyte carriage as estimated by qRT-PCR was slightly shorter in the AL group (4.5 days) than in the DP group (5.1 days). This stands in contrast to the results of a study by Sawa et al. in an area close to this one in Mbita Western Kenya, in which AL showed a threefold times faster gametocyte claearance rate than DP. This could be an early evidence of lower efficacy of AL against asexual parasites or gametocyte specifically. Clearly, this suggest more tests are required to establish AL’s superior gametocytocidal efficacy.

Submicroscopic gametocytes persisted for up to 42 days in minority of individuals, weeks after treatment initiation, indicating a prolonged gametocytaemia. Gametocyte carriage in children has been described previously [23], but still unclear whether the prolonged period could be explained by recurrent asexual parasitaemia or ACT [24]. Previous studies have shown that submicroscopic gametocytes are common in symptomatic children and can infect mosquitoes [23, 25]. Consequently, it may suggest the role of age-dependent immune suppression of gametocytaemia. The significance of infectious reservoir of malaria in general population has been described in previous studies [17, 26] but still remains less clear.

The study highlights the limitations of ACT against gametocytes. The results indicate that over 25% of the children remained gametocytaemic 5 weeks after treatment initiation. This finding has been reported elsewhere [10, 22, 25, 27] suggesting the persistence of gametocytes up to 42 days after treatment. As previously shown, submicroscopic gametocytes can persist for up to 1 month after treatment with ACT and even longer after treatment with non-artemisinin based combinations [3]. The failure of the currently used anti-malarials to clear mature gametocytes may allow onward malaria transmission week(s) following treatment in areas with intense malaria transmission. Previous studies have reported that prolonged gametocytaemia following treatment could be an early sign of the emergence of drug resistance, which is also the case in the occurrence of recrudescent infections [25, 28]. These findings provide insightful information in the future design of any anti-malarials that aims at reducing gametocytes.

A limitation of the present study was that evidence on the transmissibility of submicroscopic gametocytes to mosquitoes could not be established, because whole blood samples for the children in the study were not available for membrane feeding assays. During sample collection only, blood spot filter papers and blood smears were obtained during follow-up visits as per the protocol.

## Conclusion

The data indicate that gametocytes persisted up to 42 days after treatment in minority of individuals in both treatment arms. The study detects no significant difference between AL and DP in gametocyte clearance rate. This stands in contrast to the results of a study in an area close to this one in Mbita Western Kenya, in which AL showed a threefold times faster gametocyte claearance rate than DP that was significant. More tests are required to establish AL’s superior gametocytocidal efficacy and an additional gametocytocidal drug in combination to ACT would be useful in blocking malaria transmission more efficiently in areas where malaria endemicity is high.

## Data Availability

The dataset generated by this study is available from the corresponding author upon request.
